# 2159. Analysis of Resistance to Oral Standard-of-Care Antibiotics for Urinary Tract Infections Caused By *Escherichia coli* and *Staphylococcus saprophyticus* Collected in the United States in 2022

**DOI:** 10.1093/ofid/ofad500.1782

**Published:** 2023-11-27

**Authors:** S J Ryan Arends, Renuka Kapoor, Didem Torumkuney, Nicole E Scangarella-Oman, Rodrigo E Mendes

**Affiliations:** JMI Laboratories, North Liberty, Iowa; GSK, Atlanta, Georgia; GSK, Atlanta, Georgia; GlaxoSmithKline plc., Collegeville, Pennsylvania; JMI Laboratories, North Liberty, Iowa

## Abstract

**Background:**

Gepotidacin is a novel, bactericidal, first-in-class triazaacenapthylene antibiotic that inhibits bacterial DNA replication by a distinct mechanism of action and binding site and provides well-balanced inhibition of 2 different Type II topoisomerase enzymes. This study reports on the *in vitro* activity of gepotidacin and other oral antibiotics when tested against contemporary *Escherichia coli* and *Staphylococcus saprophyticus* clinical isolates collected from patients with UTIs for a gepotidacin uUTI global surveillance study.

**Figure:**
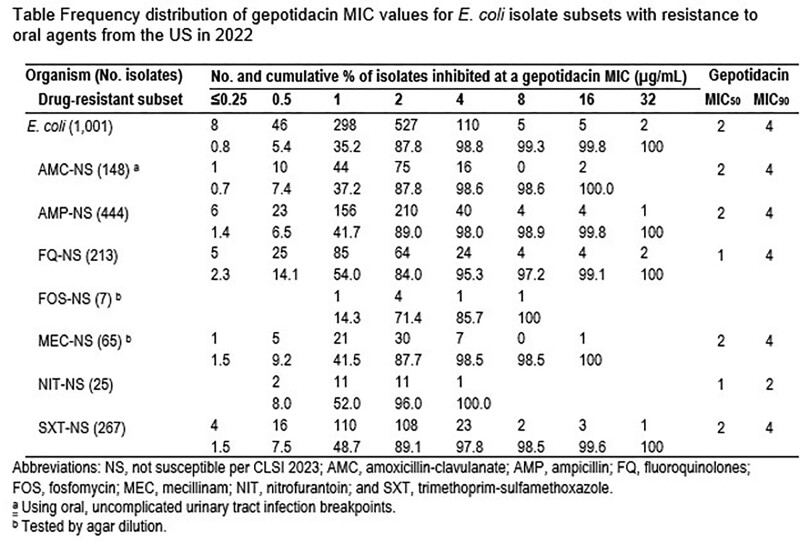

**Methods:**

A total of 1,001 *E. coli* and 92 *S. saprophyticus* isolates were collected during 2022 from 45 medical centers located within the United States. Most isolates (77%) tested were cultured from urine specimens collected from patients seen in ambulatory, emergency, family practice, and outpatient medical services. Bacterial identifications were confirmed by MALDI-TOF MS. Isolates were tested for susceptibility by CLSI methods at a central laboratory (JMI Laboratories). MIC results for oral antibiotics licensed for the treatment of uUTI and drug-resistant subsets were interpreted per CLSI guidelines.

**Results:**

Gepotidacin (MIC_50_/_90_, 2/4 µg/mL) displayed good activity against 1,001 *E. coli* isolates as 98.8% of all observed gepotidacin MICs were ≤4 µg/mL (Table). Other oral agents tested against these isolates demonstrated the following rates of susceptibility (S): amoxicillin-clavulanate (85.2% S), ampicillin (55.6% S), ciprofloxacin (78.8%S), fosfomycin (99.3% S), mecillinam (93.5%S), nitrofurantoin (97.5% S), and trimethoprim-sulfamethoxazole (73.3% S). Gepotidacin maintained similar MIC_50_ values (ranging from 1 – 2 µg/mL) and MIC_90_ values (ranging from 2 – 4 µg/mL) against these drug-resistant subsets. Against *S. saprophyticus* isolates, gepotidacin (MIC_50/90_, 0.06/0.12 µg/mL) inhibited all isolates at ≤0.12 µg/mL. Most oral agents showed >90% S against *S. saprophyticus* isolates, except for penicillin (7.6% S).

**Conclusion:**

Gepotidacin demonstrated *in vitro* activity against contemporary *E. coli* and *S. saprophyticus* from US urine isolates. This activity remained unaffected by resistance to other oral standard-of-care antibiotics.

**Disclosures:**

**S J Ryan Arends, PhD**, Cipla: Grant/Research Support|GSK: Grant/Research Support **Renuka Kapoor, PhD**, GSK: Grant/Research Support **Didem Torumkuney, PhD**, GSK: Grant/Research Support **Nicole E. Scangarella-Oman, MS**, GSK: Employee and shareholder **Rodrigo E. Mendes, PhD**, AbbVie: Grant/Research Support|Basilea: Grant/Research Support|Cipla: Grant/Research Support|Entasis: Grant/Research Support|GSK: Grant/Research Support|Paratek: Grant/Research Support|Pfizer: Grant/Research Support|Shionogi: Grant/Research Support

